# Improving the IT Experience of Resident Doctors Within West London NHS Trust: A QIP

**DOI:** 10.1192/bjo.2026.11473

**Published:** 2026-06-30

**Authors:** Saikat Roy, Mandy Bassi

**Affiliations:** West London NHS Trust, London, United Kingdom

## Abstract

**Aims:**

Poor IT experience and support are well-recognised contributors to inefficiency, frustration, and burnout among resident doctors. Within West London NHS Trust, informal feedback suggested significant difficulties with access to clinical systems, hardware availability, and IT support responsiveness, impacting both workflow and patient care. This is especially noticeable during the rotation time–February and August. This Quality Improvement Project aimed to identify the IT difficulties resident doctors were facing and to improve resident doctors’ IT experience at West London NHS Trust by addressing key barriers.

**Methods:**

Baseline data were collected using an anonymised online survey assessing access to IT hardware, login functionality, system reliability, and IT support satisfaction. Process mapping and stakeholder engagement with IT services, clinical leads, and junior doctor representatives identified priority areas for intervention. Change ideas were tested using Plan–Do–Study–Act (PDSA) cycles. Medical education manager attended the induction and ensured that all the new starters have access to the key systems–clinical system, pathology etc. Difficulties were flagged up to the ICT service.

**Results:**

In the pre-intervention questionnaire, 67% residents reported of experiencing some IT difficulties. The difficulties were with regards to accessing pathology system (ICE), hardware related, windows and NHS email log in. Key concerns around the IT experience were addressed this December, during the rotation of FY doctors. And they reported of improved experience. However, much larger number of doctors rotate in February and August. Similar induction day IT support will be made available in the first week of February. Data from this rotation will show the full impact.

**Conclusion:**

IT system’s access was made part of the induction and early detection of difficulties helped to resolve some of the issues which would otherwise impact work in the first few weeks of the rotation. During the December rotation favourable outcome was noted.

